# Targeting Oncogenic Transcriptional Networks in Neuroblastoma: From N-Myc to Epigenetic Drugs

**DOI:** 10.3390/ijms222312883

**Published:** 2021-11-28

**Authors:** Roberto Ciaccio, Piergiuseppe De Rosa, Sara Aloisi, Marta Viggiano, Leonardo Cimadom, Suleman Khan Zadran, Giovanni Perini, Giorgio Milazzo

**Affiliations:** Department of Pharmacy and Biotechnology, University of Bologna, 40126 Bologna, Italy; roberto.ciaccio2@unibo.it (R.C.); piergiuseppe.derosa2@unibo.it (P.D.R.); sara.aloisi4@unibo.it (S.A.); marta.viggiano2@unibo.it (M.V.); leonardo.cimadom@studio.unibo.it (L.C.); sulemankhan.zadran@unibo.it (S.K.Z.); giovanni.perini@unibo.it (G.P.)

**Keywords:** neuroblastoma, oncogene, tumour suppressor, regulatory network, gene expression, MYCN, CRC, HDACi, GD2, epigenetic therapies

## Abstract

Neuroblastoma (NB) is one of the most frequently occurring neurogenic extracranial solid cancers in childhood and infancy. Over the years, many pieces of evidence suggested that NB development is controlled by gene expression dysregulation. These unleashed programs that outline NB cancer cells make them highly dependent on specific tuning of gene expression, which can act co-operatively to define the differentiation state, cell identity, and specialized functions. The peculiar regulation is mainly caused by genetic and epigenetic alterations, resulting in the dependency on a small set of key master transcriptional regulators as the convergence point of multiple signalling pathways. In this review, we provide a comprehensive blueprint of transcriptional regulation bearing NB initiation and progression, unveiling the complexity of novel oncogenic and tumour suppressive regulatory networks of this pathology. Furthermore, we underline the significance of multi-target therapies against these hallmarks, showing how novel approaches, together with chemotherapy, surgery, or radiotherapy, can have substantial antineoplastic effects, disrupting a wide variety of tumorigenic pathways through combinations of different treatments.

## 1. Neuroblastoma: An Overview

Childhood and paediatric cancers are among the most relevant causes of death that affect children during the first years of life. Although researchers took into consideration several risk factors that can determine cancer development in children and adolescents, their causes are mostly unknown. Moreover, the absence of recommended screening tests able to define tumour formation and evolution was complicated by methodological difficulties related to the biological variety of these diseases, all contributing to the poor pathology outcome. Nevertheless, progresses of new high-throughput sequencing techniques and the application of genome-wide analyses made it possible to better understand the genetic backgrounds of many paediatric cancers, which influences their initiation and evolution. In this context, neuroblastoma (NB) is a clear example of how altered gene expression affects the dysregulation of crucial cellular mechanisms, such as proliferation, differentiation, chromosome stability, and self-renewal [1]. Remarkably, NB is considered the most common extracranial solid tumour identified in infancy, with 25–50 cases per million individuals. It is the first cause of death for children between one and five years of age, representing 13% of overall paediatric cancers. This neuroendocrine tumour arises in the developing sympathetic nervous system, the fallout of which is tumour development localized both in the adrenal glands and sympathetic ganglia, and it differs from other solid tumours by its biological and clinical heterogeneity, spanning from spontaneous regression to overly aggressive metastatic diseases [1].

The cellular origin of NB is not precisely identified yet; therefore, the observed clinical diversification of NB is ascribed to the disruption of the meticulously arranged process of neural crest maturation through the connection of many molecular components at various stages. The broad spectrum of NB clinical behaviour describes a challenging goal for diagnosis, prognosis, and selection of the most fruitful treatment strategy—moreover, its obstacles compare clinical trials between different studies. In 1988, an international congress was held to elaborate a system intended to help the clinical trial procedures. The result was the International NB Staging System (INSS), according to which NB can be classified into five distinct stages [2]. Stage 1 and stage 2 NB, considered low-risk tumours (>90% survival rate), are relatively small, specifically localized, not metastatic and can be entirely removed by surgery; however, stage 2 NB could be persistent after surgery, requiring further treatment with chemotherapy and/or irradiation. Stage 3 NB is an intermediate/high-risk tumour (30–50% survival rate), with metastatic infiltration in lymph nodes nearby the original onset site but not in distant parts of the body. The more aggressive Stage 4 is the high-risk tumour (<30% survival rate), with metastases spread through distinct parts of the body, including lymph nodes, liver, skin, and bone marrow. The fifth stage, 4S, is significantly different from the earlier classes; it can initially manifest typical aggressive tumours, but then it undergoes spontaneous regression with minimum treatment, or even without medical intervention (average survival rate 50–80%). This tumour is usually diagnosed via standard histology analysis and detection of unusual urinary catecholamines. At the same time, the five stages are recognized according to a series of features that include the age of diagnosis, MYCN gene amplification status, histology, and localisation of eventual metastases [2]. More than 50% of all patients are diagnosed with either stage 3 or stage 4 NB that may yield various tumour phenotypes [3]. This incredible tumour variability well reflects the genetic heterogeneity typical of this condition.

## 2. Genetic Predisposition and Chromosome Instability in NB

To date, no single genetic lesions are known to account for all NB patients. These data support the idea of NB as a spectrum of diseases rather than a single pathological condition. Nonetheless, some genetic alterations and oncogenic drivers have diagnostic and prognostic significance in specific stratification groups (Table 1) [4,5]. Although the aetiology of this disease is still not fully understood, NB can be classified as either sporadic or familial, depending on whether the mutation occurred in the patient. Indeed, the NGS (next generation sequencing) revolution has led to a deeper understanding of the NB genomic landscape. These novel approaches shed light on several aberrations already involved in the tumorigenesis and revealed new ones, spanning from gains and deletions of entire chromosomes to segmental chromosome alterations and single-nucleotide mutations. The first identified familial gene for NB was PHOX2B, whose mutations predispose to disease development in ~10% of familial cases. It encodes a master regulator transcription factor (master TF), playing a pivotal role in early embryogenesis for autonomic nervous system development [6,7,8]. Consequently, mutations in this gene are associated with a spectrum of pathological phenotypes. PHOX2B NB-specific mutations are gain-of-function, missense, and frameshift variants, mapping at 200–300 bp and 600–714 bp from the ATG start codon [9].

Despite PHOX2B, the anaplastic lymphoma kinase (ALK) gene is considered the major susceptibility gene for familial NB and the most often mutated gene in sporadic cases. ALK encodes a transmembrane receptor tyrosine kinase involved in nervous system development. It acts as an oncogene for several human tumours (such as several types of lymphoma and non-small cell lung cancer), mainly activated by chromosomal rearrangements resulting in fusion genes. Differently, the most frequent ALK alterations in NB are both sequence mutations, involving the tyrosine kinase protein domain and causing constitutive ALK activation and kinase activity, and copy number alterations, given by trisomy 2p and gene amplification [10,11]. ALK can also be affected more rarely by structural alterations resulting in N-terminal deletions, leading to a truncated isoform being constitutively activated and supporting oncogenic properties [12,13,14].

However, familial cases of NB account for only 1–2% of cases and the majority of NB are the sporadic ones. The most common and most clearly implicated genomic alteration is the MYCN gene amplification, which is present in ~18% of cases, considering WGS or WES data of 1232 NB samples from two different studies combined with data generated by the Therapeutically Applicable Research to Generate Effective Treatment initiative (TARGET, https://ocg.cancer.gov/programs/target_phs000467 (accessed on 21 June 2021 via cBio Portal for Cancer Genomic https://www.cbioportal.org/)) [15,16]. MYCN focal gain is a well-established prognostic factor for high-risk cases, marked by advanced tumour stage, high aggressiveness, and poor outcome. The origin of MYCN amplification is still unclear, but it is considered an initiating event for tumorigenesis in high-risk cases, conferring stem-like properties to MYCN amplified cells [17,18,19]. Approximately 70% of NB patients with MYCN amplification show loss of heterozygosity (LOH) at 1p36, a segmental chromosomal alteration common to different human cancers, especially the ones affecting the nervous system. The hypothesis is that this region holds several gene-dosage-sensitive tumour suppressor genes co-operating with driver mutations for oncogenesis. Regarding NB, 1p36 deletion is the most common genomic lesion in high-risk cases. Its role in tumorigenesis has been explained by in vitro studies in mouse-derived cell models, which revealed 1p36 LOH could achieve neoplastic transformation collaborating with other alterations or encourage MYCN amplification, depending on deletion sizes. Moreover, two tumour suppressor genes have been revealed. CHD5 has been proposed as a distal tumour suppressor, involved in smaller 1p36 deletions associated with MYCN single-copy NB and MYCN-amplified NB. Otherwise, ARID1A acts as a proximal tumour suppressor, promoting tumorigenesis via MYCN amplification in tumours harbouring larger 1p36 deletions [20]. ARID1A encodes a factor belonging to the SWI/SNF complexes and regulates gene expression through chromatin structure modulation. ARID1A depletion supports the adrenergic-to-mesenchymal transition by regulating enhancer-mediated gene expression, thus promoting cell invasion and tumour resistance to chemotherapy [21].

Other frequent structural variants in NB are 17q gain and 11q deletion. Gain of the long arm of chromosome 17 (17q) is the most common chromosomal rearrangement in NB, considered a characteristic sign of high-risk cases, in association with MYCN amplification and 1p deletion [22]. The hypothesis for the increased aggressiveness of 17q gain NB is that 17q gain itself could enhance genomic instability, increasing tumour cell mutational burden. Particularly, the resulting overexpression of several electron transport chain genes mapping at the 17q locus would lead to an increase in ROS production and, so, improve tumour genetic alterations, intensifying tumour aggressiveness [14]. Several genes have been proposed as 17q oncogenes, but further analysis is needed to better define 17q oncogene contribution to NB. JMDJ6 gene has been identified as an NB tumorigenesis factor, inducing reduction in NB cell proliferation and survival in vitro and tumour progression in mice when it is knocked down [23]. Using both whole-genome and RNA sequencing data, prohibiting gene (PHB) is highly expressed in 17q gain NB, enhancing tumour cell proliferation and suppressing differentiation with a novel mechanism [24]. Increased NME1 gene expression has also been associated with high-risk NB, suggesting a potential role of histidine kinase signalling in tumour pathogenesis [25]. Moreover, ALYREF gene has been proposed as a codriver factor for oncogenesis in NB by in vitro and in vivo transgenic models, co-operating with N-Myc for USP3 transcription upregulation, and thus regulating MYCN turnover [26]. However, a better definition of these genes’ involvement is still to be determined. 11q deletion is the segmental chromosomal alteration most often associated with other genomic alterations in high-risk NB, and the most identified following MYCN amplification and 17q gain. MYCN amplification and 11q deletion co-occurrence are sporadic; 11q deletion has been shown to be associated with large 2p gain, including MYCN and other genes, such as ALK [27]. This inverse correlation between deletion of 11q locus and MYCN focal gain could be due to their common effect in NB pathogenesis: both can disrupt microRNA let-7, which is considered to possess a fundamental role in tumour development. 11q deletion could be the missing aberration to acquire in order to reach the threshold for let-7 disruption in a context of insufficient MYCN copy number [28]. 11q deletion tumours are characterised by older diagnosis, more advanced disease stages, and a higher risk of relapse. Several genes involved in DNA repair mechanisms map at this locus, such as TSLC1, ATM, and H2AFX. Their missing activity caused by haploinsufficiency or inactivation of the second allele by other mutational events could explain the high chromosomal instability marking 11q del NB and the higher relapse probability and worse prognosis [27]. Chromosome 11 is also involved in other NB-relevant chromosomal rearrangements, such as translocation involving chromosome 11 and 17 (t (11;17)) and disrupting SHANK2 gene. SHANK2 is a neurodevelopmental gene encoding a scaffold protein in the postsynaptic density and was recently identified as a new tumour suppressor gene in NB. Indeed, among genes recurrently disrupted by structural variants in NB, a strong enrichment in neurodevelopmental disorders (NDDs) genes has been found, in addition to their downregulation in high-risk NB cases. Among them, SHANK2 showed the most reduced expression, and it has been proposed that its deregulation can promote NB cell dedifferentiation and poor survival. Moreover, other autism spectrum disorder (ASD) and synaptic genes, such as DLG2 (also mapping at 11q locus and frequently disrupted by translocation events in NB), have been proposed as candidate genes with a similar role in NB pathogenicity. In contrast, germline mutations associated with NDDs have been found to confer an increased risk of developing the tumour [29]. Therefore, neurodevelopmental processes seem to have a key role in NB tumorigenesis, and further investigations on these genes need to be performed.

Most NB cancer cells also show genomic alterations resulting in the activation of telomeres maintenance mechanisms, allowing tumour cells infinite proliferation and survival, consisting of the upregulation of telomerase activity or the activation of the ALT pathway. Elevated telomerase activity is associated with high-risk NB and can be due to both MYCN amplification and TERT gene rearrangements, which occur, respectively, in 40% and 20% of high-risk NB. Rearrangements in TERT upstream and downstream regions would cause TERT overexpression by genomic repositioning, acting as potent cancer driver mutations [15,30]. Instead, ALT pathway activation is principally caused by ATRX mutations, usually age-correlated, and occurs in patients older than 18 months [31]. ALT is a telomeres maintenance machinery based on homologous recombination and telomerase independence. ATRX protein belongs to a polyprotein complex involved in chromatin remodelling, nucleosome assembly, and telomeres maintenance. Loss-of-function mutations in ATRX lead to defective H3.3 deposition and replicative forks stall at the telomeric regions, inducing ALT activation for telomeres elongation. Furthermore, the second mechanism of contribution to tumorigenesis has been shown for ARTX: defective H3.3 deposition in other genomic regions, such as promoter and enhancer regions of neuronal differentiation genes, would cause their attenuated expression and proliferation of cancer cells [32].

In addition to the above-mentioned and well-known segmental chromosomal alterations, other structural variants have also been identified in NB cases, such as 3p and 4p deletions and 1q gain. However, they are relatively more rare alterations whose role has still to be clarified. Similarly, the implication of numerical chromosomal alterations (NCA) is still not well defined. It is known that gains or losses of entire chromosomes and triploidy are associated with the absence of segmental chromosomal alterations and a favourable prognosis. For example, in a recent retrospective multi-centric study, whole chromosome 19 gain has been identified in a subgroup of samples with lower tumour stages, absence of structural variants, and better outcomes. Moreover, in a previous study, whole chromosome X loss has been proposed as a new prognosis marker for NCA cases. However, few specific issues have been described, and studies are needed [33]. As briefly described above, the presence of genomic alterations in NB cells seems to be nonrandomly distributed. Instead, it appears to be under a relationship of co-occurrence and mutual exclusion. For example, MYCN amplification, TERT gene rearrangement, and ALT pathway activation caused by ATRX mutations converge on the similar cellular result (telomere length maintenance) and are mutually exclusive [15,30]. Specifically, MYCN amplification and ATRX-inactivating mutations are incompatible in all stages of NB, leading to synthetic genetic lethality due to the excessive DNA-replicative stress caused by these alterations [32]. By contrast, MYCN and TERT rearrangement relationships are not clear, and there is still no consensus among data about it. In some cases, both MYCN and TERT alterations have been identified in the same samples [14]. Differently, due to their common location at 2p, ALK amplification often shows co-occurrence with MYCN amplification. Indeed, an MYCN–ALK co-operation model based on PI3K signalling has been proposed [19]. However, from a recent analysis of WGS data from 182 diagnosis samples, no statistically significant co-occurrence has been identified between ALK and other gene alterations. The same study also confirmed the mutual exclusivity relationship between MYCN alterations and other segmental chromosome aberrations, such as t (11;17), 3p deletion, 4p deletion, and 11q deletion. Moreover, these data support the already known absence of correlation between 1p deletion and other copy number alterations, and confirm the co-occurrence between 3p deletion, 4p deletion, 11q deletion, 7q gain, and 17q gain. NB genomic alteration shows different prevalence among age: it has been proposed that the acquisition of specific oncogenic mutations could be age-correlated due to a different sensibility of developmental stage. MYCN and TERT alteration are more common in patients below five years old, while ARTX mutations are enriched in older patients. Otherwise, segmental chromosomal alteration is more common from 1.5 years old [14]. Several studies have also investigated the molecular genetic profiling of adult-onset NB, proposing that the poorer survival in an adult could be caused by the presence of different genetic alterations between paediatric and adult NB [31,34,35]. However, from a recent study investigating the molecular genetics of adult-onset NB, no differences have been identified in the type of genetic alterations between paediatric and adult NB, supposing that paediatric and adult NB differ in frequency and not to the kind of specific changes [36].

## 3. Emerging Concepts of Epigenetic Dysregulation in NB

While most of the alterations responsible for inducing the familial NB cases have been well characterised, the same cannot be said regarding the sporadic ones, which show reduced levels of genetic alterations when sequenced [43]. This fact has shifted the attention on the epigenome: the whole asset of chemical modifications targeting DNA or histone protein tails can bear regulating gene expression throughout the genome. The epigenetic changes shape chromatin conformation, thereby easing or obstructing the binding of specific factors to specific genomic sequences. Several different actors take part in this process, and they can be roughly summarized in three classes: “writers” able to label histones with post-translational modifications, “readers” which bind specifically to the labelled chromatin regulating gene expression, and “erasers” restoring the unmodified protein [44]. The concentred action of these factors generates a specific signature that can be interpreted to gain further insight into normal development and pathological contexts. Recent chromatin immunoprecipitation (ChIP) assays coupled with high throughput sequencing revealed distinct epigenetic labelling, characterising not only subtypes of NB itself [45], but also differentiating subtypes of neural stem cells (NSCs)-derived tissues, such as neuroectoderm, neural crest, and more mature neural states, thus, stating the ability of NSCs to adopt multiple fates upon commitment [46]. The epigenome is, thereby, a cell fate indicator: an observation that can be exploited to highlight alterations within chromatin structure during mammalian development [47,48]. Malignancies usually involve both the genetic and the epigenetic landscape, with nonfamilial NB cases being an exception: the poor frequency of genomic mutations states an epigenetic-mediated alteration of the transcription’s balance, leading to cancer progression via multiple ways, such as increased cell plasticity or tumour suppressor gene silencing [49].

For instance, both NSCs and embryonic stem cells (ESCs) are characterised by many genes kept transcriptionally silenced until differentiation, even though they display both permissive and repressive epigenetic marks on the relative promoters [50]. The definition of “bivalent domains” can explain this situation. Both permissive (H3K4me3) and repressive (H3K27me3) marks consent to rapidly route cells towards any fate, considering that genes enriched with H3K27me3 in ESCs include those involved in early embryonic development, organogenesis, and cell fate decisions. Genes needed to be transcribed lose most of their polycomb-mediated repressive H3K27 methylation. On the other hand, those maintained as silenced lose their H3K4 methylation and/or increase the polycomb-induced repressive epigenetic mark [50,51]. The high impact at which epigenetic regulation can influence transcriptional balance is commonly acknowledged among the scientific community. Interestingly, one of the most prominent histone methyltransferases (HMT), EZH2, a “writer” member of the polycomb repressive complex 2 (PRC2) and responsible for H3K27 trimethylation, is characterised by increased expression levels in NB [52]. In fact, several in vitro and in vivo studies reported that, in MYCN-amplified NBs, the gene promoter of EZH2 is directly regulated by N-Myc [53,54,55]. N-Myc can also directly interact with EZH2, and overexpression of EZH2 promotes an undifferentiated NB tumour phenotype associated with poor clinical outcomes [53,56].

A further example of an important chromatin-regulating actor in NB biology is the histone demethylase LSD1 (KDM1A), an “eraser” factor able to demethylate H3K4me2/me1 to unmethylated H3K4, which is associated with gene silencing. A high level of LSD1 function in an NB context correlates with a bad prognosis of patients and poorly differentiated cells (in vitro assays) [57].

Transcription factors (TFs) can also recruit chromatin-remodeller-containing repressive complexes to their target loci. Above all, N-Myc can exert repressive functions interacting with the basal transcription factor 1 (SP1). This repressive complex requires the sequence-specific transcription factor MIZ-1 to bind targets’ promoter regions and recruit other chromatin modifiers, such as the “eraser” histone deacetylases (HDAC) [58,59]. The most well-characterised repressive complexes that are known to have a role in NB development are the polycomb repressive complex 2 (PRC2), Sin3, nucleosome remodelling and deacetylase complex (NuRD), silencing mediator for retinoid and thyroid hormone receptors (SMRT), C-terminal binding proteins (CtBP), and REST corepressor (CoREST) complexes all sharing one or more protein of the HDAC family [60]. These complexes have been widely investigated in a cancer landscape, even though just a modest number of studies have drawn attention to their behaviour in NB and to how they differentially work in this context. Providing some examples, Gajer et al. [61] showed that inhibition of HAT activity in vitro and in vivo blocked NB cells growth; Chen et al. [54] demonstrated that knockdown of the PRC2 component EZH2 or its depletion upon inhibitor treatment resulted in markedly decreased NB cell viability; Yang et al. [62] proved that the silencing of the histone demethylase LSD1, a component of CoREST complexes, resulted in a reduction in cell proliferation, colony formation, migration, and invasion of NB cell lines. Despite the scarcity of information regarding how these complexes work and influence the initiation and maintenance of NB, the fact that the tumorigenic phenotype is reduced or inhibited after depletion of some of these complexes’ components represents a great suggestion of their importance in NB. Interestingly, these machineries show a high level of interconnection in terms of shared components, similar binding sites on chromatin, and downstream effects. Further investigations on these potentially druggable regulators might be fundamental for developing new therapeutic strategies to tackle down one or more critical pathways in maintaining a tumorigenic profile in an NB landscape.

## 4. NB Regulatory Networks

**Figure 1:**
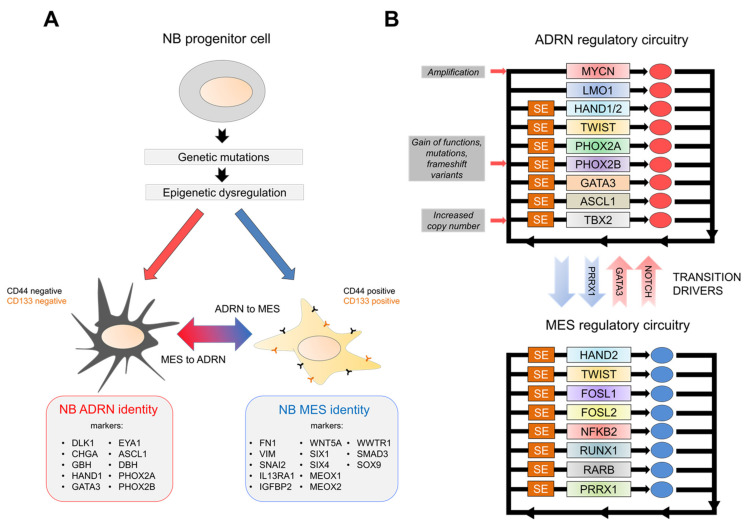
(**A**)Transition between mesenchymal (MES) and adrenergic (ADRN) neuroblastoma states depends on specific core regulatory circuitries. (**B**) Convergence of genetic mutations and epigenetic alterations results in oncogenic signalling dysregulation depending on feed-forward core transcriptional circuitries in human NBs. CRCs result in interconnected and autoregulated networks among TFs, which can drive the development of specific subtypes of NBs by establishing distinct gene expression signatures. “SE” refers to “Super Enhancer”.

While almost half of the encoded human TFs are expressed in every cell type, a restricted subgroup of them, named master TFs, can dictate the expression of cell-type-specific genes, thereby controlling transcriptional programmes in a tissue-specific fashion that will characterise the differentiated cell state (Figure 1). These core TFs are highly expressed in specific cell types, and they tend to co-occupy many enhancer and super-enhancer elements within multi-subunit protein complexes [63]. Enhancers typically drive gene transcription of multiple genomic loci, and also display consensus DNA binding sequences for several transcription factors and are found in euchromatic regions. On the other hand, super enhancers (SEs) show the same features on a higher level of magnitude: they space for a range of more than 20kb on average, have highly dense clusters of TF binding sites, and their genomic loci show outstanding levels of open chromatin histone modifications, such as H3K4me1 and H3K27ac [64]. Overall, SEs exhibit a stronger ability in activating transcription and a more substantial influence on the genes they control (Figure 1) [65].

Intriguingly, recent genome-wide H3K27ac profiling in patient-derived NB samples revealed four distinct SE-driven epigenetic subtypes, characterised by their own and specific master regulatory networks. Three of them are named after the known clinical groups: MYCN-amplified, MYCN non-amplified high-risk, and MYCN non-amplified low-risk NBs, while the fourth displays cellular features which resemble multipotent Schwann cell precursors. Interestingly, the cyclin gene CCND1 was regulated through distinct and shared SEs in the different subtypes, and, more importantly, some tumours showed signals belonging to multiple epigenetic signatures, suggesting that the epigenetic landscape is likely to contribute to intratumoural heterogeneity [45].

The establishment of interconnected and autoregulated networks among TFs represents the core transcriptional regulatory circuitry (CRC) of a cell. In cancer cells, stem and lineage-specific TFs and other epigenetic regulators are often hijacked by cancer-associated CRCs, which are activated to prevent terminal differentiation and reshape tumour cell behaviour [63,66,67,68,69]. Particularly, in NB, recent evidence reported by several groups, and, notably, by van Groningen and colleagues [70], suggested two predominant types of cell identity among neuroblastoma cell lines. These two groups are defined by highly different phenotypes and divergent gene expression profiles governed by unique core TFs and super-enhancer transcriptional networks. The first group, showing a sympathetic-noradrenergic identity, includes committed sympathoadrenal cells (ADRN), while the second group comprises multipotent undifferentiated cells with a neural crest-cell-like or mesenchymal identity, referred to as MES identity (Figure 1) [70,71,72]. In particular, it was demonstrated that around 369 genes have corresponded with ADRN mRNA expression, and 485 genes associated with MES mRNA expression in four isogenic cell lines. Interestingly, ChIP-seq analyses for H3K4me3 and H3K27ac histone modifications were performed in five ADRN and four MES NB cell lines, revealing around 276 SEs related to the ADRN type, while 286 associated with MES type.

Some essential genes, such as IL13RA1, FN1, IGFBP2, and WNT5A, were associated with MES-specific states, while genes such as DLK1, CHGA, and GBH correspond with ADRN differentiation. Further studies also revealed 20 essential MES TF genes, such as SIX1, SIX4, MEOX1, MEOX2, WWTR1, SMAD3, and SOX9, and about 18 SE-associated TF genes, such as, for example, HAND1, GATA3, EYA1, and ASCL1 [65,69,73].

Moreover, the ADRN and MES cells can transdifferentiate, with paired mesoderm homeobox protein 1 (PRRX1) being the driving factor in promoting the interconversion from the ADR to the MES module, modifying transcriptional programmes and repurposing the adrenergic super-enhancer landscape [70]. The more profound investigation of SK-N-SH neuroblastoma-derived cells was an exhaustive example to better understand the bidirectional and spontaneous potential of transdifferentiation in NB. Indeed, RNA-seq of SK-N-SH cells identified two phenotypically divergent subclones, characterised by the expression of CD44 as an MES marker [74]. Bulk RNA-seq experiments verified that CD44- and CD44+ sorted cells displayed gene expression profiles such as the ADRN SH-SY5Y and the MES SH-EP cells, respectively. The sorted ADRN/CD44- and MES/CD44+ cells can generate a mixed cell population, confirming spontaneous and bidirectional plasticity between these two states [75].

In ADRN cells of NB, downstream Notch activation [76] or overexpression of the transcription factor PRRX1 induces transdifferentiation towards an MES phenotype. Notably, this research team discovered that in vitro treatment of MES NB cells revealed more resistance to frequently used NB medicines, such as cisplatin, doxorubicin, and etoposide, than ADRN NB cells, suggesting an answer to the widespread problem of potential relapses. In addition, in vivo studies showed that PRRX1+ MES cells increase in relapsed tumours and tumours treated with conventional chemotherapy [76].

NBs belonging to the most frequent ADRN group are controlled by a set of super-enhancers, including TFs loci such as HAND2, PHOX2A, PHOX2B, GATA2, GATA3, and the ALK oncogene locus [71]. On the other hand, the MES group, significantly associated with relapsed cases, is driven by a CRC module, including AP-1 transcription factor family (FOSL1 and FOSL2), NFKB2, RUNX1, RARB [45], and PRRX1 [77] as major drivers, conferring NCC-like identity. The TFs TWIST and HAND2 are known to be bound to both cell states’ regulatory sequences [77].

Several TFs govern the gene expression programmes of neuroblastoma, notably including different CRC transcription factors, such as PHOX2B, HAND2, GATA3, ASCL1, ISL1, and TBX2, which show a clustered binding across open chromatin regions of their regulatory sequences, as well as those of the other CRC partners and those of many other driver master regulators, including MYCN and ALK. This signature array is either unique to NB or only minimally shared with other tumour types [72].

NB’s dependence on these master regulators has been proven and confirmed by several research groups via several different approaches, such as transient siRNA-mediated knockdown, footprinting, HiChIP experiments, and functional genomics studies with CRISPR–Cas9 screenings. Indeed, depletion of one of these genes resulted in a reduction in cell proliferation, induction of apoptosis, and a concomitant decrease in each CRC gene expression level, demonstrating their interdependent expression [45,71,72]. In addition, the KD of a subtype-related master regulator, specific for ADR or MES identity, strongly correlated with a higher sensitivity of the associated subtype, supporting the notion that these master TFs are required for maintaining a particular neuroblastoma cell state (Figure 1). However, despite considerable efforts, it still remains largely unknown how distinct cell identities can influence neuroblastoma’s tumour initiation, progression, and relapse, as well as the molecular events that drive NB development, which are still unclear [45,78].

## 5. N-Myc and Other Master Regulators: Oncogenic Drivers in NB Progression

In the context of NB master regulators, N-Myc plays a crucial role, since its gene amplification is associated with poor prognosis and advanced tumour stages, and is one of the initiating events driving the transformation and progression of high-risk NBs [19,79,80].

As a pivotal oncogenic transcription factor, N-Myc, along with its partner MAX, can activate or repress many genes, thereby orchestrating expression programmes of several targets that cannot be ascribed to a single regulatory pathway. When deregulated, N-Myc governs the cis-regulatory landscape of NB. Among its targets, it co-occupies the same regulatory regions bound by adrenergic CRC TFs, further promoting the transcription of genes induced by CRC members and reinforcing their expression as well in a model defined by Zeid et al. as “enhancer invasion” [77]. Consequently, loss of N-Myc leads to a reduction in global gene expression levels within the cellular transcriptome and, particularly, of its directly targeted tumour-related genes [81], indicating its determining role in maintaining both normal and altered NB regulatory networks. For this reason, N-Myc may act as a general transcriptional amplifier or, otherwise, as a CRC member [72].

ChIP-seq analyses produced by Zeid and colleagues showed different N-Myc binding profiles: in one case, its classical target genes exhibit the classical promoter occupancy, while, in some others, N-Myc shows a more spread promoter and enhancer binding. The latter shape was proved to be associated with crucial neuroblastoma-associated genes, suggesting that only deregulated N-Myc invades pre-established and preacetylated enhancers in order to amplify tissue-specific gene expression and drive oncogenic transformation [77].

In MYCN-amplified NBs, N-Myc is stabilised by several mechanisms and has a leading role in regulating different downstream pathways and proteins, notably including the anaplastic lymphoma kinase (ALK). ALK can be coamplified along with MYCN, and its gain-of-function mutations can potentiate MYCN oncogenic activity [82]. N-Myc and ALK co-operation seems to be due to ALK-mediated activation of RET and MAPK/RAS/PI3K-dependent signalling [19].

The development of MYCN-amplified NBs is also sustained by the action of other protein interactors, such as Aurora A (AURKA), a kinase that has been demonstrated to stabilise the N-Myc protein, inhibiting its degradation. AURKA may inhibit neuroblasts’ cell-cycle exit during embryonic and early postnatal development, thereby contributing to NB formation. Notably, AURKA and N-Myc are connected through a positive feedback loop: AURKA is highly expressed in MYCN-amplified NBs, while N-Myc stability is enhanced by this kinase [83].

The action of TWIST1, an already mentioned CRC TF, also sustains MYCN’s enhancer axis in MYCN-amplified NBs. TWIST1 and N-Myc recognise similar CANNTG E-boxe DNA sequences and, in addition, their binding sites on enhancers strongly overlap; this suggests crucial oncogenic co-operation between these two TFs in promoting neuroblastoma tumorigenesis and in driving enhancer-dependent gene expression [77]. Additionally, the chromatin regulator WDR5 has been shown to be needed for MYC recruitment on chromatin and, more specifically, to form a protein complex with N-Myc, leading to H3K4 trimethylation and activation of N-Myc targets. Remarkably, the repression of WDR5 resulted in a reduction in NB cell growth and apoptosis. Hence, uncovering this WDR5-N-Myc relationship could be useful for developing new therapies against MYC-driven tumours [84,85].

Among all the factors taking part in NB tumorigenesis, recent genomic and functional investigations highlighted BARD1 [32,86], LMO1, and ASCL1 [81] as important actors in neuroblastoma tumorigenesis as well.

Genome-wide association studies (GWAS) identified common variations at the BARD1 locus to be highly associated with aggressiveness of high-risk neuroblastoma [32,86]. The expression of the full-length isoform of BARD1 has been demonstrated to prevent malignant transformation of NB cells, negatively correlated with high-risk neuroblastoma development. This isoform heterodimerises with BRCA1, and it is required for BRCA1 relocation and its known tumour-suppressive function as a guardian of genetic stability. This evidence indicates BARD1 as a tumour suppressor gene. In contrast, the opposite effect was instead ascribed to cancer-associated BARD1 shorter isoforms: their interaction with the Aurora kinase family antagonises the functions of the full-length isoform, thereby defining them as one of the oncogenic drivers of NB carcinogenesis [32,86].

Recent studies by Wang et al. revealed the implication of LMO1 as a major predisposition gene in NB oncogenesis. LMO proteins operate in NB cells as additional transcriptional cofactors that function as adapters to form complexes between DNA-binding proteins, such as basic helix–loop–helix (bHLH) proteins or GATA proteins. LMO1 acts as an oncogene that collaborates with N-Myc, causing rapid cellular proliferation and arrest of neuroblasts’ differentiation into chromaffin cells or sympathetic ganglia. Although the oncogenic pathways downstream of LMO1’s transcriptional regulation are unknown, ChIP-seq and RNA-seq analyses revealed that a critical target regulated by LMO1 is ASCL1, a gene encoding a bHLH TF. The regulatory elements of this gene are also bound by all members of the adrenergic neuroblastoma CRC; moreover, ASCL1 and LMO1 proteins can cobind to the enhancers responsible for the regulation of CRC TFs genes, and to the regulatory regions of their target as well. This characteristic occupancy makes them, respectively, a member and a coregulator of the ADRN neuroblastoma CRC [81].

## 6. Transcriptional Dysregulated Programmes and Promising Therapeutic Approaches

**Figure 2:**
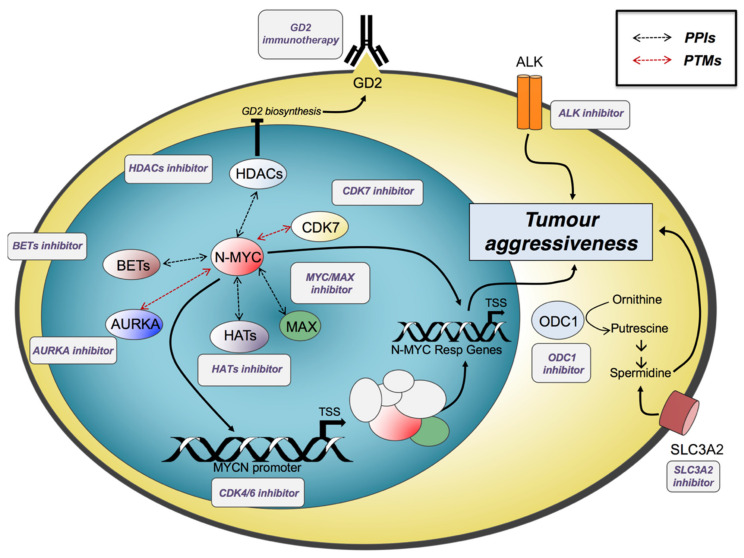
Targeting neuroblastoma oncogenic pathways: N-Myc pathways can be targeted by a direct or indirect approach. Direct targeting can be achieved by blocking the N-Myc/MAX interaction, while indirect targeting can be obtained by inhibiting enzymes involved in N-Myc post-translational modifications (PTMs) or protein–protein interactions (PPIs). Furthermore, HDAC inhibition induces higher expression of GD2, a crucial target for immunotherapy. The N-Myc-responsive gene product ODC1 can be targeted to inhibit spermidine biosynthesis, involved in tumoral aggressive phenotype. Spermidine uptake from the extracellular environment can be blocked by inhibiting the SLC3A2 transporter. ALK inhibitors are successfully used in ALK-mutated NB.

The description of the NB landscape through advances in DNA, RNA, and epigenetic profiling reveals the complexity of this pathology [16,87,88]. In this regard, it is not surprising that NB research has constantly increased over the years, showing how the extreme variety of genetic and epigenetic backgrounds, mixed with multiple levels of regulatory network regulations, reflect a challenging tumour to investigate. To date, treatment of NB high-risk patients includes an intensive chemotherapy regimen with cisplatin, vincristine, carboplatin, etoposide, and cyclophosphamide (COJEC), followed by resection surgery and myeloablative therapy in combination with haematopoietic stem cell reinfusion and local radiation therapy [89]. The relevance of specific targeted therapy in NB could be crucial considering the standard strategies’ weakening approach in treating high-risk patients and the extreme cancer heterogeneity, which spans from spontaneous regressions to metastatic and aggressive diseases (Figure 2) [90].

Several biological and genetic markers of this tumour have been understudied to help diagnosis and prognosis, giving relevant insights on the molecular landscape of NB and attention to specific factors. Indeed, the dysregulation of gene expression programmes, biochemical cascades, and metabolic pathways control the aggressiveness of NB, shedding light on some crucial components capable of being directly or indirectly targeted. Activating ALK mutations and N-Myc overexpression were shown to be the most influential de novo oncogenic drivers. Indeed, the regulatory networks dependent on N-Myc and ALK are considerably involved in maintaining the proliferative phenotype and blocking differentiation pathways in neural precursors, as demonstrated by in vitro and in vivo experiments [18].

### 6.1. Targeting N-Myc and Its Regulatory Networks

For instance, the N-Myc-dependent regulatory network drives the malignancy and maintenance of stem-like state by activating the expression of genes involved in metastasis, such as integrins α1 and β1, the FAK protein, and metalloproteinases, self-renewal and pluripotency, such as KLF2, KLF4, and LIN28B, survival, and angiogenesis [91,92,93,94,95]. Thus, novel efforts are converging on the investigation of new methods to target MYC to indirectly achieve antitumour effects by disrupting its oncogenic programme’s key components. The potential of selectively inhibiting N-Myc would be the most effective approach to counteract advanced forms of NB. Indeed, since the high frequency of MYCN amplification in cancer and its role in driving and promoting tumorigenesis, as well as its space–temporal restricted expression during embryo development, precise N-Myc targeting would certainly result in successful therapeutics to support NB treatment (Figure 2) [18,96]. However, the extreme variability in cancer mutations and the presence of homologous forms of Myc proteins are still profoundly affecting the process of selective drug design. Specific N-Myc inhibitor therapy still remains poorly explored [97]. To overcome this issue, the scientific community focuses on alternative approaches that aim to control N-Myc-mediated transcriptional activation and its regulatory networks. Once chromatin undergoes “writers” and “erasers” labelling, it will gain or lose the ability to bind to the “readers”. One outstanding example is the bromodomain-containing protein BRD4, which recognises and binds histones with acetylated lysine residues [98]. BRD4 interacts with the positive elongation factor (P-TEFb) complex as part of the general transcription machinery. It thereby regulates gene expression by participating in the transcription preinitiation complex assembly [99].

Indeed, N-Myc-mediated transcriptional regulation is promoted mainly by the association with bromodomain and extra terminal (BET)-containing proteins, which work as chromatin “readers” by binding to acetylated lysine residues and helping transcription. The bromodomain-containing protein 2 (BRD2), BRD3, and BRD4 are of great relevance. Several analyses showed that the application of the BET inhibitor JQ1 downregulates N-Myc transcriptional signatures, lowering MYCN expression, thus increasing the survival percentage in both xenograft and transgenic murine models of NB (Figure 2) [100].

Although not yet approved by the American agency of Food and Drug Administration (FDA), the application of BETi seems to be one of the most promising approaches to treat NB patients with MYCN amplification. Further proof of this was provided by developing new drugs, such as birabresib (MK-8628—formerly known as OTX015—an orally bioavailable small molecule that prevents BRD2/3/4 from binding to acetylated histones. Recently, Henssen et al. showed that BRD4 specifically occupies N-Myc targets and other genes associated with super-enhancers, and that OTX015 specifically disrupts BRD4 binding to chromatin in NB MYCN-driven murine models, leading to significant survival advantage compared with untreated controls (Table 2) [101]. This study established the therapeutic efficacy of the BET inhibitor OTX015 in preclinical NB studies. Moreover, it confirmed the effectiveness of this drug in phase I trials in adult haematological malignancies (NCT01713582) and solid tumours (NCT02259114), as well as for GSK525762, another BETi under phase I clinical trial for solid tumours, including NB [102]. As an amplifier of active transcription, the modern concept of Myc proteins is constantly evolving compared to the commonly held conclusion that Myc co-ordinates the transcription of distinct groups of genes. This event can be possible since N-Myc can interact with a plethora of proteins, allowing the regulation of several central control points of gene transcription, such as promoter binding, epigenetic modifications, initiation, elongation, and post-transcriptional processes [58,77,103,104].

RNA polymerase II (RNA Pol II) transcriptional activation is regulated by a specific set of cyclin-dependent kinases (CDKs), including CDK7 (cyclin-dependent kinase 7), a crucial component of the transcription initiation factor TFIIH that phosphorylates RNA Pol II to start transcription. In 2014, Chipumuro et al. reported that a covalent inhibitor of cyclin-dependent kinase 7 (CDK7), THZ1, was found to disrupt the transcription of MYCN-amplified NB cells selectively, leading to global repression of N-Myc-dependent transcriptional amplification and induction of tumour regression in mice models (Figure 2, Table 2) [105]. The substantial selectivity of this compound for cells with MYCN amplification may be attributable to the reduced expression of super-enhancer-associated oncogenic drivers, including the same N-Myc. Combinatorial therapy with THZ1 and the tyrosine kinase inhibitor (TKi) ponatinib and lapatinib, as well as with the HDACi Panobinostat, synergistically induced NB cell apoptosis, leading to NB tumour regression [23,106].

These novel therapeutic approaches are gaining even greater importance considering the effect on the regulation on the CRC and global gene expression, confirming how particularly JQ1 and THZ1 injection can rapidly decrease the expression of CRC mRNA levels after just one hour of treatment in MYCN-amplified NB cells. The expression level of each of the CRC transcription factor genes was dramatically downregulated by the combination of JQ1 and THZ1, with more restricted consequences regarding either drug alone. These results underlined the impact of JQ1 and THZ1 combination treatment in MYCN-amplified NB, although the broad implication of combining transcriptional disruption is still not fully understood [72]. In line with these novel pharmacological strategies, recent studies shed light on the CDK9/2 inhibitor CYC065 (fadraciclib) contribution, resulting in selective loss of nascent MYCN transcription (Table 2). MYCN loss sensitises cells to apoptosis following CDK2 inhibition by selectively targeting NB cells characterised with MYCN amplification, confirming the pivotal role of the crosstalk between the components of the transcriptional machinery [107].

#### 6.1.1. Inhibition of the N-Myc/MAX Interaction

N-Myc is a nuclear intrinsically disordered protein that can exist in distinct complexes within the same cell by interacting with hundreds of components to keep the cell identity [104,217,218].

All the Myc proteins are basic helix–loop–helix/leucine zipper (bHLH/LZ) transcription factors capable of dimerising with the partner MAX (MYC-associated factor X) to regulate up to 10–15% of all genes. These data assume additional relevance within MYCN biology in NB models, considering how MAX can instruct transcriptional programmes that either reinforce or weaken the oncogenic process enacted by N-Myc [219]. Thus, the N-Myc/MAX heterodimer-controlled inhibition is an attractive approach to counteract the oncogenic regulatory network triggered by MYCN [220]. In 2002, a 7000 peptidomimetic compound screening was performed to select novel candidates capable of preventing the dimerisation between Myc and MAX (Figure 2). This analysis identified IIA6B17 and IIA4B20, two small molecules that exert a strong inhibitory effect on Myc–MAX dimerisation and DNA binding, characterised by a lower IC50 for Myc compared to the homologous transcription factor Jun [221]. The ground-breaking work of Berg et al. began the development of novel therapeutic interventions to inhibit the MYCN-mediated oncogenic programme. The identification of new drugs, such as 10074-G5, 10058-F4, KJ-Pyr-9, the Myc inhibitor 361 (MYCi361), and the novel Peptomyc’s Omomyc-based therapy (OMO-103) brought new hope for the fight against NB disease (Table 2) [88,101,222,223,224]. Remarkably, just in March 2021, OMO-103 was announced to have obtained approval from the Spanish Agency of Medicines and Medical Devices for conducting a phase I/II clinical trial, proving the efficacy of this innovative approach (NCT04808362).

#### 6.1.2. Targeting N-Myc Stability

As for c-MYC, the N-Myc protein degradation is mainly induced by the ubiquitin–proteasome system [225]. The discovery of new components affecting the N-Myc protein stability aroused great interest concerning the possibility of developing novel treatments against many MYC-driven tumours. In this framework, AURKA inhibition is making inroads as a promising alternative approach in preclinical models of NB. N-Myc is usually stabilised by direct interaction with AURKA, preventing proteasomal degradation dependent on the SCF-FBXW7 E3 ubiquitin ligase (Figure 2) [83]. Confirming the importance of this topic, the AURKA inhibitor MLN8237 (also known as alisertib) combined with irinotecan and temozolomide chemotherapy is under clinical assessment for multiple-cancer relapsed NB (NCT01601535) (Table 2) [182]. The characterisation of a new class of conformation-disrupting inhibitors of AURKA that destabilise interactions between AURKA and N-Myc is enjoying great popularity, proving to be another promising strategy in the next future operations [226]. As for AURKA, WDR5 is emerging as a novel promising MYC vulnerability in cancers [227]. Following this approach, many other drugs, such as the PLK1 inhibitor BI 2356, the HAUSP inhibitor P22077, and the PA2G4 inhibitor WS6, are providing the basis for drug design of small molecules targeting Myc and N-Myc binding partners in malignancies driven by MYC family oncoproteins, representing new alternative forms for the treatment of high-risk NB [203,228,229].

### 6.2. Targeting Polyamine Metabolism

MYCN-amplified NBs show deregulation of several enzymes involved in polyamine metabolisms, such as ODC1, SRM, SMS, AMD1, OAZ2, and SMOX [230]. Polyamines are essential polycations that sustain Myc functions through ionic and covalent activities. The decreased levels of intracellular polyamines stimulate checkpoints that limit proliferation, while enhanced polyamine synthesis supports oncogenic proliferation (Figure 2) [231]; this pathway has aroused attention as a therapeutic target in cancers and other hyperproliferative diseases. Moreover, polyamines are involved in several biological processes, and one of the most crucial ones is spermidine. Spermidine is needed for hypusinilation of the translation elongation factor eIF5A, engaged in translating genes containing specific aminoacidic repeats. Hypusinilated eIF5A is essential for translation of genes encoding for proteins involved in the cytoskeletal-associated process, RNA splicing and turnover, DNA binding and transcription, and cell signalling [232]. Notably, ODC1, a key enzyme in polyamine metabolism which converts ornithine to putrescine, is a direct transcription target of N-Myc [230,233]. ODC1 is druggable by difluoromethylornithine (DFMO), which recently completed a phase II clinical trial. DFMO treatment after completion of first-line therapy was associated with improved event-free and overall survival compared to controls treated at the same institutions of this clinical trial [233]. The importance of ODC1 was recently underlined by Gamble et al., showing how a G316A promoter single nucleotide polymorphism (SNP) differentially affects ODC1 expression, as well as MYCN-mediated ODC1 transactivation of the E-box region and MYCN oncogenic processes in NB cells in vitro. The underlying molecular mechanism revealed that the A allele had decreased affinity for the N-Myc protein, indicating that the region surrounding the E-box is critical in modulating ODC1 transcriptional function and an influence on DMFO response (Table 2) [234]. Chemoresistance to DFMO may be due to the upregulation of transporters that drive polyamine uptake from the extracellular environment. SLC3A2 is a transporter able to drive intake polyamines into NB cells, and its inhibition by AMXT 1501 in combination with DFMO was effective in treating NB in mouse models (Table 2) [196]. Combination therapy of AMXT 1501 and DFMO treatment is currently in phase I trial for solid tumours (NCT03536728).

### 6.3. Targeting CDK4/6 and PI3K/AKT/mTOR

On the other hand, N-Myc dysregulation can influence cell cycle progression by upregulating several genes, such as cyclin D2, E2F proteins, CDK4/6, and CDC2, resulting in the inactivation of genes involved in the G1 phase and DNA replication (Figure 2) [235,236]. These data assume additional relevance in preclinical studies and clinics considering the promising effects of CDK4/6 inhibitors on NB and other paediatric cancers [137,138,139].

One of the principal pathways accountable for pushing the malignant transformation and drug resistance in solid tumours is the phosphatidylinositol 3-kinase (PI3K)/AKT/mTOR pathway. PI3K is a class of molecules divided into three categories based on structure, regulation, and function [237]. This pathway is taking particular attention after the pioneering work of Opel et al., which showed, in 2007, how the activation of the PI3K–AKT–mTOR pathway is a common event in NB tumour samples. This pathway drives the phosphorylation of AKT at threonine 308 (T308) and/or serine 473 (S473), an event that correlates with less overall survival in NB patients with MYCN amplification, 1p36 chromosomal alterations, which drives general decrease in event-free survival [238]. The specific mechanisms by which the PI3K/AKT/mTOR pathway is activated in NB are still not fully understood. Accordingly, the actual hypothesis is that PI3K/AKT/mTOR pathway activation in NB occurs through various mechanisms. Indeed, some scientists advised that the reduced expression of the tumour suppressor PTEN may diminish PI3K signalling through reducing negative regulation on the p110 subunit encoded by the PIK3CA gene [239]. This is supported by several pieces of evidence of reduced PTEN protein levels evaluated in immunohistochemistry, together with an increase in downstream targets of the PI3K pathway in NB [240]. Recent investigations have also underscored mutations in the ALK gene, such as ALK F1174L, capable of increasing, together with N-Myc, the PI3K/AKT/mTOR pathway in a subset of NB [241]. Hence, this phenomenon seems to also be related to TrkB/brain-derived neurotrophic, IGF, EGF, PDGF, and VEGF receptor signalling pathways [242,243,244,245,246]. To date, the PI3K/AKT/mTOR pathway is arousing interest as a novel target for potential novel therapeutic approaches. For example, the selective insulin-like growth factor 1 (IGF-1) receptor inhibitor picropodophyllin (PPP) has been shown to prevent AKT activation and, consequently, suppress cell proliferation in NB cells. In addition to that, the dual mTORC1–mTORC2 inhibitor AZD8055, the phase I trial SF1126, the phase I/II trial copanlisib (PI3K inhibitor), the phase I/Ib trial perifosine (AKT inhibitor), and many other compounds are emerging as great opportunities to fight this overly aggressive disease, marking the significant importance of this molecular pathway (Table 2).

### 6.4. Targeting ALK

Nowadays, mutated ALK is one of the few directly targetable main oncogenes in NB [247]. As discussed previously, the most common ALK mutations found in NB are amplification and point mutation, driving its hyperactivation through autophosphorylation of the tyrosine kinase domain [108]. Several ALK inhibitors have been developed for cancer therapy, and they have been classified as first-, second-, and third-generation ALK inhibitors. Here, we report three representative examples of drugs belonging to each of these categories (Figure 2).

First-generation ALK inhibitor crizotinib is a small-molecule adenosine triphosphate (ATP)—a competitive inhibitor of ALK kinase activity. Crizotinib showed satisfactory results in some paediatric cancers carrying gain-of-function ALK mutation. However, in a phase I clinical trial involving 11 NB patients with known ALK mutation, only one patient with Arg1275Gln mutation experienced complete regression and two patients with Arg1275Gln and Phe1174Leu mutation experienced stable disease [248]. This is consistent with further in vitro data showing enhanced growth inhibition of crizotinib in NB cell lines with Arg1275 mutation than cell lines carrying other mutations [108]. Indeed, crizotinib showed minimal effects on ALK Phe1174 mutations, one of the most common ALK mutations seen in NB. The second-generation ALK inhibitor alectinib induces apoptosis in both ALK wild-type and ALK mutant NB cells, and it is even more effective when combined with HDAC inhibitor vorinostat (Table 2) [113]. However, to our knowledge, there are no clinical studies on NB with this drug. Third-generation ALK inhibitor lorlatinib is one of the most promising molecules for the treatment of ALK-mutated NB. It showed good efficiency in vitro and in vivo in NB cells carrying Arg1275Gln, Phe1174Leu, and Phe1245Cys mutations, and it is currently in phase I study [109] (NCT03107988 NCT04753658). Notably, a recent case report of a patient carrying ALK Phe1174Leu mutation showed a complete response to lorlatinib but, unfortunately, relapsed after 13 months from the treatment (Table 2) [117].

### 6.5. Epigenetic Therapies

In compliance with the low frequency of recurrent somatic mutations, NB is mainly characterised by the dysregulation of a multitude of genetic and epigenetic mechanisms, leading patients to poor outcomes [249]. This cancer model is an outstanding candidate for discovering new epigenetic therapies to overcome drug resistance. As previously discussed, the perturbation of the histone acetylome plays a key role during the whole process of tumorigenesis; indeed, various active molecules targeting histone acetylation regulatory enzymes, such as bromodomains (BRDs), histone deacetylases (HDACs), and histone acetyltransferase (HATs), have been developed to recover abnormal histone acetylation levels due to the dysregulation of these regulatory networks, and some of them were already positively evaluated in clinical trials also (Figure 2) [60].

Based on the vast heterogeneity of the HDAC classes, many studies on different pathologies describe a full-blown uneven cellular response to both the specific and nonspecific HDAC inhibition [196,250]. Consistent with transcriptional addiction, the selective disruption of CRC was accomplished by targeting the acetylation in cancer. Concerning NB, various investigations confirmed the therapeutic potential of HDACi in terms of efficacy, toxicity, and pharmacokinetics (Table 2). One of the key examples is valproic acid, capable of taking the cells to a marked inhibition of cell proliferation, induction of differentiation, suppression of the Warburg effect, and apoptosis [176,251,252]. Consistent with the idea of combinatory drug treatments, valproic acid showed increased therapeutic potential in combination with other drugs, such as the DNA-damaging molecule ellipticine [253] (PMID: 29304031), the COX-2 inhibitor celecoxib, the clusterin inhibitor OGX-01170, the angiogenic inhibitor ABT-510, or the more commonly used etoposide and cisplatin, thus proving synergistic effects leading to tumour growth impairment (Table 2) [177,253,254,255,256]. A synthetic lethal screening against MYCN-amplified NB revealed that the HDACi vorinostat (also known as SAHA) induces dramatic cell death combined with the proteasome inhibitor bortezomib (BTZ) in part through synergistic activation of BAX. This high-throughput screening was performed using a library containing 938 FDA-approved drugs for candidates that elicit synthetic lethal effects in MYC-driven NB cells. The combination resulted in marked tumour suppression in vivo, supporting dual proteasome/HDAC inhibition as a potential therapeutic approach for MYC-driven cancers [183]. Vorinostat is a selective class I and II HDAC inhibitor in a clinical trial for NB and various cancer treatments. Several studies suggested that vorinostat administration results in G2/M phase arrest, which activates the intrinsic apoptotic pathway [257]. It was also advertised to reduce VEGF secretion, suggesting a potential antiangiogenic effect [258]. Recent scientific articles strongly suggested that vorinostat administration, combined with GD2 immunotherapy, is even more effective in suppressing NB growth in the aggressive orthotopic model, resulting in higher animal survival rates, thus supplying a solid rationale for clinical testing in NB patients [184,259]. Moreover, vorinostat can also boost up the antitumour effect of other molecules, such as fenretinide, the pan-CDK inhibitor flavopiridol, and the ALK inhibitor alectinib (Table 2) [260,261,262].

Finally, recent studies suggested how HDAC inhibition can drive the enhancer remodelling and suppression of oncogenic super-enhancers by disrupting the three-dimensional structure of the chromatin looping and by depleting transcription factors on the same DNA sites [100,263].

### 6.6. Immuno Cell Therapy: Targeting GD2

Immuno-targeting MYCN-amplified NBs are challenging, since N-Myc is involved in downregulation of the major histocompatibility complex (MHC) class I antigen expression, leading to escape from cytotoxic T cells and interferon-mediated immune response [264,265]. The relationship of MYCN with the tumour immune microenvironment has only begun to be explored [264]. The tumour microenvironment of MYCN-amplified NBs contains a significantly lower number of immune cells compared to the MYCN-nonamplified counterpart [266]. For these reasons, the authors of the above-mentioned review describe MYCN-amplified NBs as “cold” and immune exclusive, while MYCN-nonamplified are referred to as significantly inflamed or “hot” [264]. Based on this evidence, a more profound comprehension of the microenvironment’s role in NB disease could drive novel strategies for the cure of this childhood malignancy (please refer to PMID: 32722460). In fact, the lack of antigen presentation due to MYCN overexpression has been successfully circumvented by developing antibodies against the targetable ganglioside GD2 [267].

Gangliosides are modified sphingolipids highly expressed from cancer cells. Generally, gangliosides are not considered for target therapy because they are also found in healthy tissues. Exceptionally, the ganglioside GD2 is highly expressed on the surface of NB cells, and its expression in normal tissue is limited at a relatively low level to neurons, skin melanocytes, and peripheral nerve fibres (Figure 2) [268]. Moreover, GD2 is virtually expressed in all NBs, regardless of the grade and staging of the tumour [269,270,271]. These characteristics make GD2 an exciting target for immunotherapy. Currently, dinutuximab is the only monoclonal antibody approved by FDA and EMA for anti-GD2 immunotherapy in NB. It is used in combination with granulocyte-macrophage colony-stimulating factor (GM-CSF), interleukin-2 (IL-2), and 13-cis-retinoic acid (RA) for the treatment of high-risk NB patients who achieve at least a partial response to prior first-line therapy [272]. Dinutuximab is a chimeric antibody (mouse–human) based on the structure of the murine monoclonal antibody 14G2a. The structural characterisation of the 14G2a antibody–antigen complex led to the generation of a specific single-chain variable fragment (ScFv), a useful tool for developing new biotechnological therapies [273]. However, limiting factors in GD2 immunotherapy are probably due to the molecular mechanisms regulating GD2 expression in NB that are not yet fully understood (Table 2) [165,166,274]. Recent evidence suggests that the enzymes involved in GD2 biosynthesis are controlled by another druggable target in NB: HDACs activity. Kroesen and colleagues demonstrated that expression of GD2 is enhanced by HDACi vorinostat, pan HDACi givinostat, class-I inhibitor entinostat, and an HDAC1,2-specific inhibitor. Their data highlighted that vorinostat induces higher GD2 expression, increasing GD2 synthase protein levels without altering its mRNA levels in the NB cell line, suggesting that HDACs may act at the post-translational level of this enzyme [184]. Moreover, the same research group found that cells that were treated with vorinostat, together with the cell-permeable sialic acid Ac5Neu5Ac, increased the expression of sialyltransferases ST3GAL5 and ST8SIA1, generating GM3 and GD3 gangliosides, the necessary precursors for GD2 synthesis [275]. The combined treatment with engineered sialic acid and HDACi may further increase the efficacy of current and future GD2-targeted immunotherapy in NB patients.

## 7. Conclusions

NB is one of the topmost common neurogenic extracranial solid cancers occurring in childhood and infancy. The extreme heterogeneity of this pathology is still considered a significant challenge to overcome and assumes a relevant aspect for developing novel therapeutic strategies. Several new pieces of evidence show the critical level of plasticity of this neoplasia, unveiling the role of numerous layers of regulation that drastically influence patient outcomes. This review aims to provide a comprehensive blueprint of the transcriptional regulation bearing NB initiation and progression, unravelling the complexity of this pathology’s key oncogenic and tumour suppressive regulatory networks. Some interesting points emerged from our analysis.

Firstly, while most familial NB cases are characterised by a “specific” subset of genetic alterations, the same cannot be said regarding the sporadic ones. Hence, the vast majority of NB patients are distinguished by multiple levels of epigenetic alterations. It is important to underline how this sort of dichotomy between these two subgroups of NBs is just apparent; indeed, the most severe NB cases are characterised by both genetic and epigenetic aberrations, resulting in widespread deregulation of gene expression profiles and disruption of these signalling networks that control proliferation and cellular response. These data lay the groundwork for evaluating the whole contribution of these intricate networks of regulation, which influence the destiny of the patients like a single far-reaching entity. Secondly, most NBs include tumour cells with diverging gene expression profiles: the two main subgroups are the undifferentiated mesenchymal (MES) cells and the committed adrenergic (ADRN) ones, which can interconvert and resemble from different lineage differentiation stages. Once again, NB’s identities are not governed by small groups of macromolecules, but by a set of core regulatory circuitries of lineage transcription factors associated with super-enhancers and several protein complexes that drastically influence the fate of the cell. This new evidence demonstrates that cancer cells’ diverging transcriptional states match with the normal lineage development stages. In particular, lineage transcription factors can lead to transdifferentiation via remodelling the epigenetic and transcriptional landscapes, mimicking the natural interconversion. All this information assumes additional relevance considering the different response of ADRN and MES cells to therapeutics, giving more strength to studying these complex grids of regulation. Third, the more profound investigation of these new vulnerabilities paves the way for developing novel therapeutic approaches, bypassing the difficulties in obtaining compounds against molecules complex to target, such as several master TFs like the same N-Myc protein. The significance of a network-based targeted therapy in neuroblastoma could be of great importance considering the standard strategies’ weakening approach in treating high-risk patients (general surgery, chemotherapy, radiotherapy), distinguished by metastatic and aggressive tumours. Although the interplay between regulatory networks and oncogenesis has been unequivocally described, more in-depth research into their role in modulating cancerous-unrelated signalling pathways still needs to be carried out. According to the novel findings summarised above, discovering this intricate reticulum of interactions represents invaluable predictive factors for early-onset disease detection.

## Figures and Tables

**Table 1:** Leading genetic alterations in NB.

Gene Name	CHr.	Alteration Type	Known NB Variants	Mutation Effect	MYCN Status °	References
Single-Gene Alterations						
ALK	2p23	Point mutation (missense)	Met1166Asn; Ile1171Asn/Thr; Phe1174Leu/Cys/Ile/Val/Ser ^§^; Leu1240Val; Phe1245Ile/Cys ^§^; Arg1275Gln/Leu ^§^	Gain of function	Amp + non-Amp	[37], #
		Amplification	-		Amp	[38]
		Translocation/ Deletion	-		-	[12,13]
ATRX	Xq21.1	Point mutation (nonsense)	Glu285 *; Glu990 *; Leu1645 *	Loss of function	non-Amp	[32], #
		Point mutation (frameshift deletion)	Phe2113Serfs *9			
PHOX2B	4p13	Point mutation (missense and frameshift)	Several variants clustered at 200–300 bp and 600–714 bp from the translation start codon	Gain of function	-	[9]
TERT	5p15.33	Upstream/downstream regions rearrangements	-	Gain of function	non-Amp	[15,30]
**Segmental Chromosomal Alterations**						
-	1p36	Deletion	-	Loss of function	Mostly amp	[20]
-	17q	Gain	-	Gain of function	Amp	[39]
-	11q	Deletion	-	Loss of function	non-Amp	[27]

MYCN status: Amp = presence of amplified MYCN; non-Amp = non-amplified MYCN. * indicates translation termination codon in nonsense and frameshift variants. § Amino acid residue identified as a recurrent hotspot (statistically significant) in a population-scale cohort of tumour samples of various cancer types, using methodology based in part on Chang et al. [40] and according to cancerhotspots.org. # cBio Portal for Cancer Genomic [41,42]. NB data come from 1459 patients/1472 samples obtained by combining four different studies [15,16,43] and data generated by the Therapeutically Applicable Research to Generate Effective Treatment initiative (TARGET, https://ocg.cancer.gov/programs/target_phs000467 (accessed on 21 June 2021 via cBio Portal for Cancer Genomic https://www.cbioportal.org/)). Sequence variants are reported according to Human Genome Variation Society (HGVS).

**Table 2:** List of chemical compounds targeting multiple regulatory networks in NB. Inhibitors are classified based on the molecular target/mechanism, preclinical (ncbi), and clinical (FDA) status relative to NB and some paediatric solid tumours updated to 2021. All the references are rereferred to in the “reference” section in the main manuscript.

Compound	Target/Mechanism	FDA Approval	Clinical Trial Status (2021)	References
Crizotinib	ALK	NCT03126916 NCT01606878 NCT00939770 NCT03107988 NCT01121588	Phase III Phase I Phase I/II Phase I Phase I	[108,109,110,111,112]
Alectinib		---	---	[113,114,115,116]
Lorlatinib		NCT04753658 NCT03107988	Observational Phase I	[117,118]
Alisertib	AURKA	NCT01601535 NCT02444884 NCT01154816	Phase I/II Phase I Phase II	[119,120,121,122,123,124,125]
JQ1	BRD2/3/4	---	---	[101,126,127,128,129,130]
OTX015		NCT01713582 NCT02259114	Phase I Phase I	[101,131,132]
GSK525762		NCT01587703	Phase I/II	[133,134]
Palbociclib	CDK4/6	NCT03526250 NCT03709680 NCT03155620	Phase II Phase I Phase II	[135,136]
Ribociclib (LEE011)		NCT01747876 NCT02780128 NCT03434262	Phase I Phase I Phase I	[137,138,139,140]
Abemaciclib (LY2835219)		NCT02644460 NCT04238819	Phase I Phase I	[141]
THZ1	CDK7	---	---	[23,105,106,127]
CYC065 (fadraciclib)	CDK9/2	NCT02552953	Phase I	[107]
Carboplatin	DNA synthesis	Approved	Approved	[142,143,144,145,146,147]
Cisplatin	DNA/RNA synthesis	Approved	Approved	[148,149,150]
Cyclophosphamide	DNA replication/RNA synthesis	Approved	Approved	[151,152,153]
Doxorubicin	DNA/RNA synthesis	Approved	Approved	[154,155,156,157,158,159,160,161]
Etoposide	DNA synthesis/Topo II poison	Approved	Approved	[142,144,162,163,164]
GD2 immunotherapy	GD2 ganglioside	NCT01822652 NCT01460901 NCT01576692 NCT01953900 NCT02100930 NCT01953900 NCT04539366	Phase I Phase I Phase I Phase I Phase I Phase I Phase I	[165,166,167,168,169,170]
PU139	HAT	---	---	[61]
PU141		---	---	[61]
P22077	HAUSP	---	---	[171]
Panobinostat	HDAC	NCT04897880	Phase II	[23,172,173,174,175]
Valproic acid		NCT01204450	Phase I	[176,177,178,179,180,181]
Vorinostat (SAHA)		NCT01019850 NCT03332667 NCT03561259 NCT01208454 NCT02035137 NCT02559778 NCT01132911 NCT01163383 NCT04308330 NCT00217412	Phase I Phase I Phase II Phase I Phase II Phase II Phase I Phase II Phase I Phase I	[182,183,184,185,186,187,188]
10058-F4	MYC/MAX heterodimer inhibitor	---	---	[189]
10074-G5		---	---	[189,190]
IIA6B17		---	---	[191]
MYCi361		---	---	[192]
Omomyc (OMO-103)		NCT04808362	Phase I/II	[193,194,195]
DFMO	ODC1	NCT02395666 NCT01586260 NCT04301843 NCT01059071 NCT02679144 NCT02139397 NCT02030964 NCT02030964	Phase II Phase II Phase II Phase I Phase II Phase I/II Phase I Phase I	[196,197,198,199,200,201,202]
WS6	PA2G4	---	---	[203]
AZD8055	PI3K/AKT/mTOR pathway	NCT01316809 NCT00973076 NCT00731263 NCT01194193	Phase I Phase I Phase I Phase I	[204,205,206,207]
Perifosine		NCT01049841 NCT00776867	Phase I Phase I	[208,209,210]
Picropodophyllin (PPP)		NCT01721577 NCT01725555 NCT01062620	Phase I/II Phase I Phase I	[211]
SF1126		NCT02337309 NCT00907205	Phase I Phase I	[212]
AMXT 1501	SLC3A2	NCT03536728	Phase I	[196,202]
Lapatinib	TK	---	---	[106,213]
Ponatinib		---	---	[106,214,215,216]

## Data Availability

Not applicable.

## Abbreviations

The following abbreviations are used in this manuscript:

ADRN	Adrenergic
BET	Bromodomain and Extra Terminal
bHLH-	basic Heli Loop Helix
CDK	Cyclin Dependent Kinase
ChIP	Chromatin Immuno Precipitation
CRC	Core Regulatory Circuitry
EMA	European Medicines Agency
ESCs	Embryonic Stem Cells
FDA	U.S Food and Drug Administration
GWAS	Genome-Wide Association Studies
HAT	Histone Acetyl Transferase
HDAC	Histone DeAcetylase
HMT	Histone Methyltransferase
INSS	International NB Staging System
KD	Knock Down
KO	Knock Out
MES	Mesenchymal
NCA	Numerical Chromosomal Alterations
NSCs	Neuronal Stem Cells
NGS	Next Generation Sequencing
NB	Neuroblastoma
SE	Super Enhancer
RA	Retinoic Acid
TARGET	Therapeutically Applicable Research to generate effective Treatment
TF	Transcription Factor
WGS	Whole Genome Sequencing
